# Transdisciplinary Obesity Prevention Research Sciences (TOPRS) Curriculum Increases Knowledge About Complex Causes and Consequences of Obesity for Undergraduate Students

**DOI:** 10.3389/fpubh.2019.00232

**Published:** 2019-08-20

**Authors:** Barbara H. Fiese, Amber Hammons, Brenda Koester, Gabriela L. Garcia, Loran Parker, Dorothy Teegarden

**Affiliations:** ^1^Family Resiliency Center, University of Illinois at Urbana-Champaign, Champaign, IL, United States; ^2^Child and Family Science, California State University, Fresno, CA, United States; ^3^Education Development Center, Chicago, IL, United States; ^4^Evaluation and Learning Research Center, Purdue University, West Lafayette, IN, United States; ^5^Department of Nutrition Science, Purdue University, West Lafayette, IN, United States

**Keywords:** obesity curriculum, flip-the-classroom, active learning, undergraduate education, public health

## Abstract

Most researchers and public health officials would agree that the causes and consequences of obesity are complex and multi-faceted. However, curricula designed to address these complexities are limited and often guided by a single discipline. The purpose of the Transdisciplinary Obesity Prevention Research Sciences (TOPRS) program was to develop a “flip-the-classroom” curriculum on obesity prevention across multiple disciplines such that students would gain an appreciation of the complex origins of obesity. The curriculum is based on the 6 C's model (cell, child, clan, community, country, culture) that proposes a cell-to-society approach to obesity. Twenty video micro-lectures were developed and students were tested on content knowledge pre- and post-viewing. The curriculum was administered at three university sites to 74 undergraduate students across 23 declared majors from 2014–2016. There were significant gains in knowledge about the causes and consequences of obesity. Recommendations are offered to adopt this curriculum in undergraduate and other educational settings.

## Introduction

There is little disagreement that the origins of the obesity epidemic are multifactorial and progress toward a solution has been stubbornly slow. In a recent call to action for improved health care training and prevention of obesity, Dietz et al. noted that medical education and health professional training lags behind in understanding stigma associated with obesity, working on interdisciplinary teams, and modifying lifestyles (1). Others have noted that medical students have somewhat unidimensional views of the causes of obesity with over one quarter endorsing purely physiological causes and close to a quarter not endorsing causes outside of overeating and physical activity (2). Most expert panels recommend multi-pronged approaches to prevention and intervention that involve multiple disciplines working as a team and across sectors (e.g., healthcare, education, government) in order to effect the most long lasting change at a population level (3–5).

Although the call for more comprehensive and interdisciplinary approaches has been made for over a decade, educational programming has not kept pace with scientific thinking on the topic, and most public health professionals are not trained in transdisciplinary approaches (6). Currently, most educational efforts in obesity prevention and treatment occurs at the postdoctoral level in specialty programs such as those offered through Nutrition Obesity Research Centers (NORC) funded by the National Institute of Diabetes and Digestive and Kidney Diseases. There are a few doctoral level programs such as the Illinois Transdisciplinary Obesity Prevention Program (I-TOPP) (7) and the Pennsylvania State University Childhood Obesity Prevention Graduate Training Program funded by the United States Department of Agriculture, National Institute of Food and Agriculture. Rarer yet are interdisciplinary programs at the undergraduate level. A program that exposes undergraduate students to the complexities of obesity has the potential to increase the scientific workforce as well as raise awareness of the next generation of parents and public health professionals. Adding obesity as topic at the undergraduate level also has the potential to effect career choices and practice in the future.

There is increasing interest in promoting transdisciplinary education as a means to address major public health issues. Lawlor et al. describe a MPH program organized around the tenets of transdisciplinarity, including an applied problem driven approach that recognizes the multiple factors that contribute to a public health problem (8). A key component of transdisciplinary team science is the ability to work collaboratively in an integrative process across disciplines to address a common research problem (9, 10). To date, the majority of efforts to train scientists in this approach have been directed toward graduate and post-graduate level students (11). There are however, exceptions at the undergraduate level (12). In this report we describe an undergraduate transdisciplinary curriculum addressing the complexity of obesity. We provide evidence that the curriculum and its hands-on problem-solving format increased knowledge about the complexity of the causes and consequences of obesity as well as research skills.

### Pedagogical Framework

**Figure 1:**
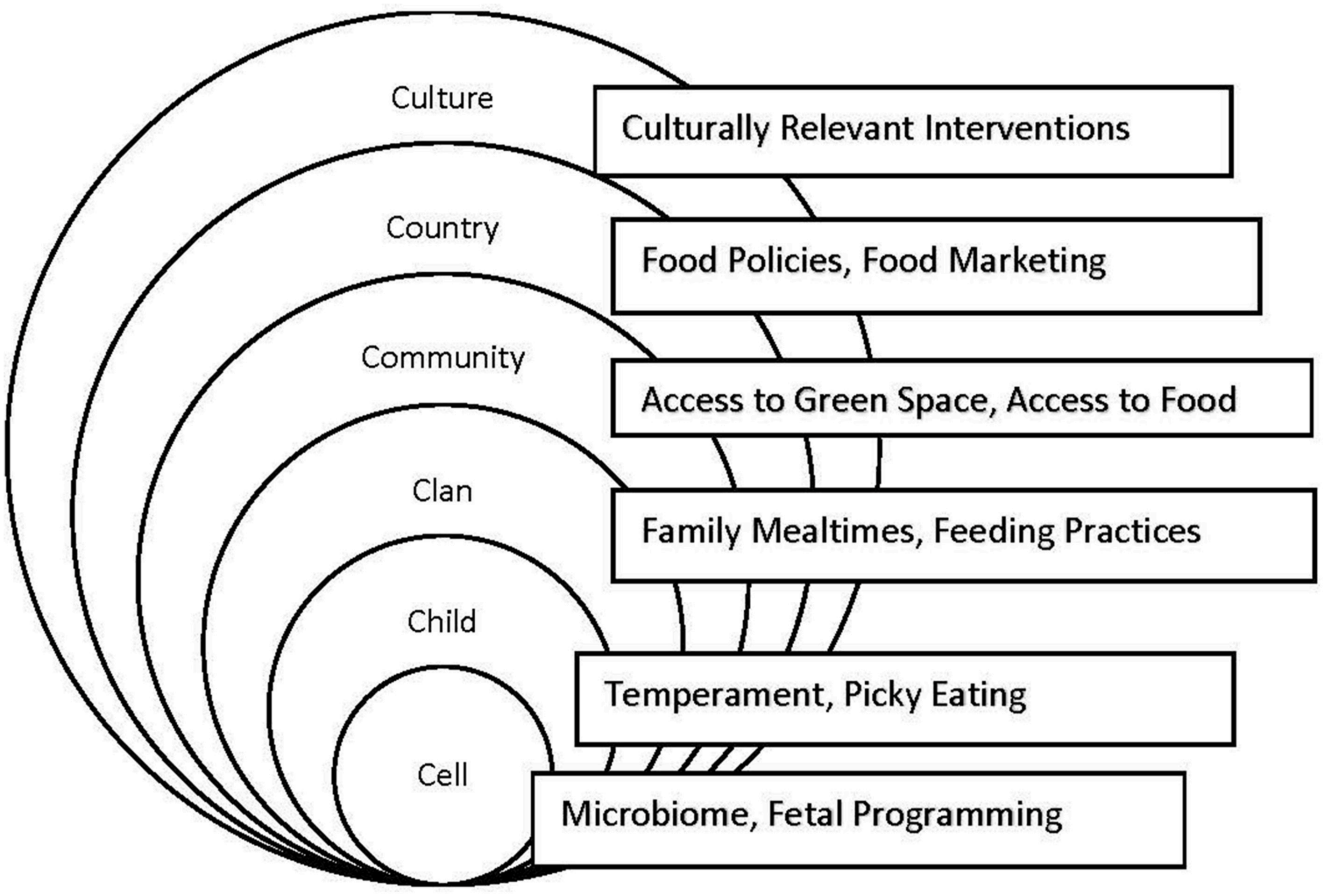
Six C's theoretical model that guided curriculum.

The curriculum was designed to take a cell-to-society approach to the causes and consequences of obesity. Using the 6 C's socio-ecological framework (13, 14) the curriculum addresses the epidemiological, biological, genetic, family, community, policy, and cultural factors that contribute to the incidence and prevalence of obesity. For example, the biological micro-lectures included overviews of genetic contributions, fetal programming, and the role of the gut microbiome. Family and child factors included picky eating, feeding practices, family mealtimes and sleep. Community factors included the built environment and physical activity as well as neighborhood influences. Policy issues were discussed, including access to food, food advertising, and food policies. Culturally relevant interventions were also discussed. In addition there were modules on the chronic health conditions associated with obesity including cardiovascular disease, diabetes, bone health, and cancer (see Figure 1 for a depiction of the theoretical model and sample curriculum topics for each ecology). The entire curriculum can be accessed at https://stemedhub.org/groups/toprs.

We applied a flip-the-classroom approach with hands-on learning activities. Several educators and professional organizations have called for a more active hands-on approach to learning at the undergraduate level (15, 16). A flip-the-classroom approach allows instructors to use valuable class time for application of content material while out of class time is spent viewing videos and reading literature that focus on content. Flipped classroom instruction has proven to increase student engagement (17), academic performance (18), and critical thinking skills (19). In our case, it was also possible to provide content from experts across multiple disciplines without relying on in-person lectures. It took close to 2 years to develop the curriculum materials, engage the experts in recording the micro-lectures, and refining the instructor handbook. However, once the materials were developed they minimized the time of the instructor for course preparation outside of updating the relevant literature and refining hands-on-learning activities.

### Learning Environment

We chose to develop the curriculum for undergraduate students who were from a variety of majors. The knowledge base of most undergraduate students about the complex origins of obesity is extremely limited. To date, most of the extant research has focused on single issues such as calories (20) or sugar sweetened beverages (21). If we are to train the next generation of scientists and public health professionals about the complexities of the causes of obesity, then it is incumbent to begin at an earlier point in the education process than medical or graduate studies. We purposely designed the curriculum to be attractive to students across multiple majors and most appropriate for students beyond their sophomore year.

The curriculum was delivered at three different sites across a period of 2 years. We deliberately created the curriculum to be flexible such that instructors could pick and choose modules to fit the needs of their particular institution- thereby avoiding some of the pitfalls of transdisciplinary education. The sites differed by the duration of the course and the direct involvement of students in research. Site 1 (Illinois) structured the course across two semesters and all students were engaged in a research project that culminates in presenting at an undergraduate research symposium at the end of the academic year. Site 2 (Purdue) offered the course in the fall following an intensive research experience in the preceding summer and through the fall semester. Site 3 (California State University, Fresno) offered the course for one semester and involved students indirectly in research through research simulations in class-based interdisciplinary teams. The class was offered across 2014–2016. Analyses was conducted throughout 2016 and into 2017.

Students watched weekly micro-lectures online and outside of class. Class time was reserved for discussion of the material and activities in interdisciplinary teams. For example, during the week on pediatric nutrition and breastfeeding, students gathered into their interdisciplinary teams and discussed the Surgeon General's Executive Summary-Call to Action to Support Breastfeeding. In these teams, students chose three of the recommended actions and designed programs and/or campaigns to implement the action. They then discussed how these actions fit within the 6 C's model of childhood obesity. One of the main projects of the course included preparing and presenting research posters. Students involved directly in research presented on their research projects while those in the indirect research experience class designed posters based on an obesity-related literature review. Students integrated theories, methodologies, and new concepts from the various disciplines in their posters.

An external evaluation team was responsible for evaluating the gain in content knowledge and confidence in research skills. Surveys were administered online pre- and post-course. The addition of an external evaluation team allowed for the cross-site evaluation and reduced the likelihood that students would respond more (or less) favorably to their own instructor. In this report we summarize the evaluation findings describing content gain and confidence in research skills pre-class and post-class participation.

## Method

### Participants

Across a period of 2 years, 74 students enrolled in the TOPRS course across the three sites. The majority of the students were women (91.9%). The students were relatively diverse in terms of race and ethnicity (38% White, 26% Asian/Pacific Islander, 19% Hispanic/Latino, 7% African American, and 10% other). The majority of the students were between 18 and 21 years of age (81.7%) with the remainder between 22 and 25 years (16.9%) and one student reported being 25 years of age or older. The students were primarily juniors and seniors (29.7% 3rd year, 31.1% 4th year) with the remainder 1st year (6.8%), 2nd year 20.2%), or 5th year (12.2%). The students listed a total of 23 different disciplinary majors ranging from Biochemistry to Speech and Hearing Sciences. A listing of majors and percentages of students within each discipline is presented in Table 1.

**Table 1:** Students' disciplinary majors.

**Disciplinary major**	**Frequency**	**Percent enrolled**
Biochemistry	1	1.4%
Chemistry	2	2.7%
Child Development	13	17.8%
Community Health	3	4.1%
Communication	1	1.4%
Dietetics	6	8.0%
Doctor of Pharmacy	1	1.4%
Economics	1	1.4%
Family and Consumer Sciences	2	2.7%
Food Science and Human Nutrition	5	6.8%
Human Development and Family Studies	4	5.5%
Integrative Biology	3	4.1%
Interdisciplinary Health	8	11.0%
Kinesiology	2	2.7%
Liberal Studies	1	1.4%
Molecular and Cellular Biology	4	5.5%
Neurobiology	1	1.4%
Pharmaceutical Science	1	1.4%
Pre-Nursing	5	6.8%
Psychology	1	1.4%
Social Work	1	1.4%
Sociology	2	2.7%
Speech and Hearing Sciences	3	4.1%
Undeclared	2	2.7%

### Procedure

Students were administered online surveys prior to the beginning of the class and at the end of the semester. The surveys assessed content knowledge about the topics taught in the class, as well as, student self-assessment of knowledge about scientific research, and self-assessment of research skills. The content varied somewhat from year 1 to year 2 as modules were added on biological causes and chronic health conditions associated with obesity. The Institutional Review Boards at all three universities approved this evaluation.

### Measures

Content knowledge was assessed through investigator developed measure designed to reflect the content of each micro-lecture. In year one three questions were generated per topic. In year two five questions were generated per topic. Answers to the questions were not part of the student's grades, and they were aware of this. Knowledge of scientific research was assessed using an instrument adapted from Seymour et al. (22). Student research skills were assessed using a research skills scale adapted from Kardash (23).

### Analytic Plan

Paired sample *t*-tests were conducted to assess changes pre-post participation in the class. Individual item analysis was also conducted to identify patterns of change to inform future curriculum development.

## Results

**Figure 2:**
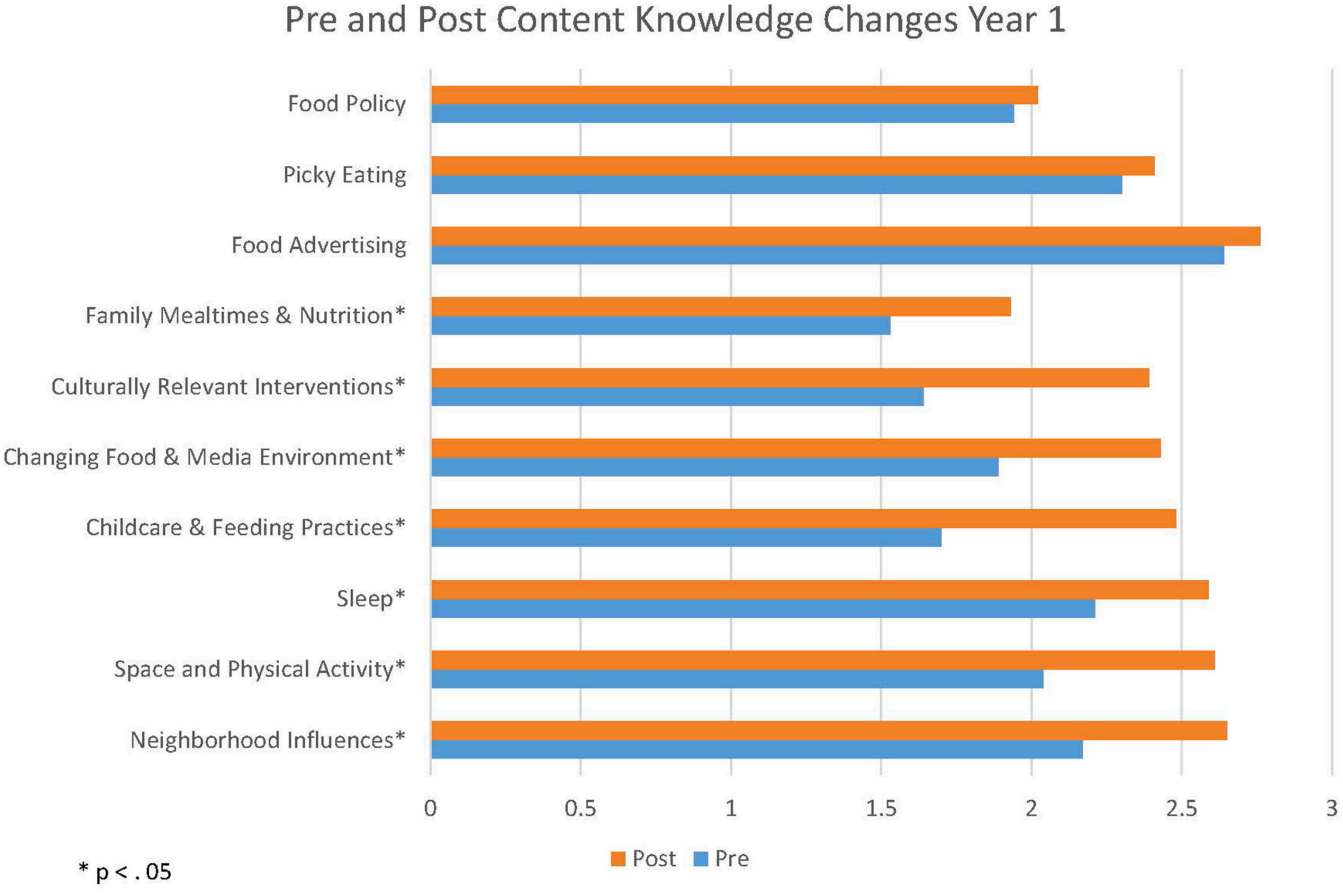
Change in content knowledge Year 1. *Means were statistically significant between pre- and post-surveys (*p* < 0.05).

There was a significant increase in content knowledge in Year 1 in seven of the 10 modules (see Figure 2). Students gained knowledge about the neighborhood influences, the role of the built environment and physical activity, sleep, childcare, and feeding practices, media, culturally relevant interventions, and family mealtimes. There were no significant gains in understanding of food advertising, picky eating, or food policy from before the class to after the completion of the course.

**Figure 3:**
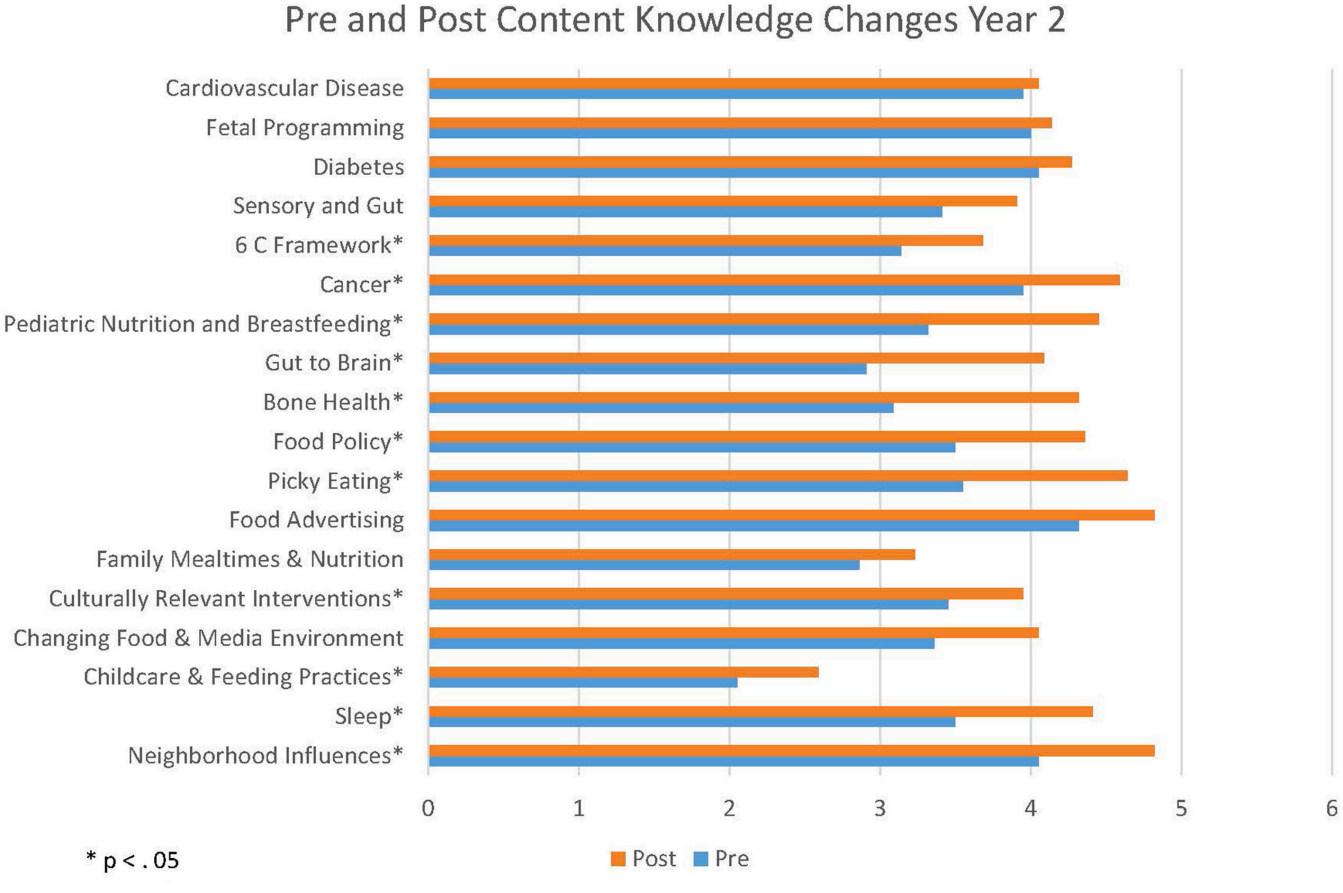
Change in content knowledge Year 2. ^*^Means were statistically significant between pre- and post-surveys (*p* < 0.05).

In year two, the students evidenced significant gains in knowledge in 14 of the 20 modules (see Figure 3). There were significant increases in knowledge about bone health, the gut to brain axis, pediatric nutrition and breastfeeding, picky eating, sleep, food policy, genetic influences, culturally relevant interventions, brain health, cancer, the 6 C framework, and child care and feeding practices from before the class until after the class ended.

Students gained significantly in their understanding of research and their research skills. Student self-assessment of their understanding of “nature of the job as a researcher” (*t* (71) = 8.225, *p* = .000) and their understanding of the “research process” (*t* (71) = 6.255, *p* = .000) displayed significant gains. At the beginning of the course, the majority of students reported a “weak” or “average” understanding of the research process (70.3%) and the nature of the job as a researcher (78.4%). By the end of the course, an overwhelming majority of the students reported having a “Strong” or “Very Strong” understanding of the research process (69.4%), and the nature of the job as a researcher (70.8%).

## Discussion

Obesity is a complex public health concern that requires transdisciplinary approaches for solutions. However, few scientists are explicitly trained in team science approaches. Rarer yet are programs at the undergraduate level that expose students to interdisciplinary and transdisciplinary methods. In this report, we documented knowledge gains and increased confidence in research skills in an undergraduate course delivered across three different sites. Overall, students gained content knowledge about the complex causes, consequences, and correlates of obesity. Further, they gained appreciation of the research process and their own confidence as researchers. In this discussion, we highlight some of the lessons learned from this evaluation and future directions for course development.

Across two evaluation points (Years 1 and 2) we were able to demonstrate that students gained content knowledge about the role that sleep, childcare and feeding practices, neighborhood influences, and culturally relevant interventions might play in obesity. Modules only evaluated once (Year 2) also showed significant gains in knowledge in the 6 C Framework, Cancer, Pediatric Nutrition and Breastfeeding, and Gut to Brain. Modules that evidenced significant gains in knowledge 1 year but not on another included Food Policy, Picky Eating, Family Mealtimes & Nutrition, and the Changing Food & Media Environment. We can only speculate about the different pattern of results. For those modules that evidenced consistent significant gains, our impression is that these are areas that students had little baseline knowledge and stood to gain the most information. Anecdotally, students were fascinated with the role that sleep played in risk for obesity. They also tended to personalize this factor in relation to their own health. In addition, they had little exposure to factors such as child care and the potential role that feeding practices in child care might play in health.

Even though we were able to show gains in some content areas- the overall purpose of the course is also to expose the students to the research process. Using valuable class time and a hands-on approach actively engaged students in critical thinking about how to develop research questions and the limits of scientific evidence. The students gained not only content knowledge about the causes and consequences of obesity but also the research process and the role of a researcher. Because they were assigned to work on teams, they also gained valuable experience from diverse backgrounds as to how the research process unfolds and how to consider multiple perspectives.

Some of the challenges to teaching the TOPRs course included students feeling that the course and research projects (for the two classes that included direct research experiences) were too separate and needed better integration. In Krettek and Thorpenberg's review of transdisiciplinary higher education, one of their suggestions for effective transdisciplinary teaching is that instructors across varied disciplines work together to design their lectures (6). The TOPRS curriculum does this in the micro-lectures, but future courses may take this a step further by having the researchers that are working directly with students on research projects come to a class and get the entire class involved in a discussion around the specific research project. Setting aside time for students to discuss their specific research projects as well as allocating time for practicing research poster presentations might also help students to see the intersection of the course content and direct research experiences. We encourage interested instructors to visit the website to determine the suitability and usefulness of the materials for their own instructional needs https://stemedhub.org/groups/toprs.

A report commissioned by the National Academy of Medicine Roundtable on Population Health Improvement recommends that interdisciplinary training be provided at multiple levels, beginning with an investment at the high school and undergraduate levels as a way of building a foundation of basic skills, competencies, and knowledge of population health concepts (9). The 2015 report lists three categories of competencies for training in population health science: knowledge, interdisciplinary skills, and knowledge exchange, and that these competencies can be achieved through immersion in an interdisciplinary environment, mentoring, and being part of an interdisciplinary team. The TOPRS undergraduate curriculum aims to provide a basic foundation for this type of training, as a central goal of the program is to actively teach interdisciplinarity. The three classes that participated in this program experienced the interdisciplinary environment, mentoring, and experience on an interdisciplinary team in varying degrees.

In two of the courses, the students participated in a hands-on research experience working on interdisciplinary teams. In the other course, students did not participate in research directly but engaged in exercises, and class activities on interdisciplinary teams designed to simulate working on research teams to solve problems related to obesity. The mentoring aspect also varied as the students working on research projects with faculty had more access to mentors in this capacity, but the instructor in the other course served as a mentor as well, but to a lesser degree. Students have fewer opportunities to get involved in research at the teaching university than at the research university, and this was the first intensive exposure to research for many of the students. As such, many of the students used the instructor as a mentor, asking questions about research and several later signed up to work on a research project with the instructor.

We recognize that this report is not without limitations. The students enrolled in the courses across sites were primarily female. Thus, further work needs to be done to consider whether the format and topic of the course appeals equally to men and women.

In conclusion, although each class experience was different, all resulted in gains in knowledge content as well as appreciation for the research process. These results are promising because the curriculum can be replicated and adapted to fit the needs of particular types of schools and students can be expected to leave the course with a basic foundation in interdisciplinary and transdisciplinary methods as applied to an important and urgent public health problem. Programs such as TOPRS can provide students exposure at the undergraduate level to transdisciplinary concepts and methodology as well as give them team building experience, which is a first step in effective collaborative science.

### Permission to Reuse and Copyright

All of the material is original and does not require permission.

## Data Availability

The evaluation data is available upon reasonable request from the authors.

## Author Contributions

BF, AH, DT, and BK originally developed the curriculum. LP and GG designed the measurement protocols. GG conducted the evaluation. All authors contributed to the writing and review of the manuscript. GG conducted the analyses presented in the manuscript.

Portions of these findings were presented at The Obesity Society as a poster in 2016. This manuscript is not currently under review at any other source.

### Conflict of Interest Statement

The authors declare that the research was conducted in the absence of any commercial or financial relationships that could be construed as a potential conflict of interest.
